# Prediction of individual differences in risky behavior in young adults via variations in local brain structure

**DOI:** 10.3389/fnins.2015.00359

**Published:** 2015-10-07

**Authors:** Zahra Nasiriavanaki, Mohsen ArianNik, Abdolhosein Abbassian, Elham Mahmoudi, Neda Roufigari, Sohrab Shahzadi, Mohammadreza Nasiriavanaki, Bahador Bahrami

**Affiliations:** ^1^Medical Faculty, Shahid Beheshti University of Medical SciencesTehran, Iran; ^2^School of Mathematics, Institute for Research in Fundamental SciencesTehran, Iran; ^3^School of Cognitive Sciences, Institute for Research in Fundamental SciencesTehran, Iran; ^4^Medical Faculty, Tehran University of Medical SciencesTehran, Iran; ^5^Department of Biomedical Engineering, College of Engineering and School of Medicine Wayne State UniversityDetroit, MI, USA; ^6^Department of Cognitive Neuroscience, University College LondonLondon, UK

**Keywords:** Voxel Based Morphometry (VBM), risk taking behavior, balloon analog risk task (BART), anterior insula

## Abstract

In recent years the problem of how inter-individual differences play a role in risk-taking behavior has become a much debated issue. We investigated this problem based on the well-known balloon analog risk task (BART) in 48 healthy subjects in which participants inflate a virtual balloon opting for a higher score in the face of a riskier chance of the balloon explosion. In this study, based on a structural Voxel Based Morphometry (VBM) technique we demonstrate a significant positive correlation between BART score and size of the gray matter volume in the anterior insula in riskier subjects. Although the anterior insula is among the candidate brain areas that were involved in the risk taking behavior in fMRI studies, here based on our structural data it is the only area that was significantly related to structural variation among different subjects.

## Introduction

Uncertainty about the future is a fact of life. Any future event arising from our own deeds or completely out of control, can be conceived of as a form of gamble which may improve or impair one's well-being by some probability distribution. Looking at future in terms of probability distributions permits quantifying the degree of uncertainty in various forms that are collectively known as the measures of risk (Schultz, 2010). In several situations, our actions can increase or decrease the risks which we face. For instance, trying a new brand of coffee can be interpreted as avoiding risk or seeking risk, respectively.

Contextual factors that modulate one's attitude toward risk have been studied extensively in economics and psychology (Tversky, 1979, 1992; Damasio, 1996). For instance, framing choices in terms of the associated loss or gain (Tversky and Kahneman, 1981; De Martino et al., 2006) or expressing their relation to the status quo (Fleming et al., 2010) as well as several other contextual factors can change one's willingness to engage in a risky behavior. However, substantial inter-individual differences have also been observed among (human as well as non-human) decision makers in their risk attitude (Fecteau et al., 2007; Rao et al., 2008; Jentsch et al., 2010). After all, some individuals are more likely to buy a lottery ticket with their Saturday morning newspaper than others. These trait-like inter-individual variations can operate in parallel to the contextual factors mentioned above and they contribute to substantial variability in risk-ridden behavior (Elke and Weber, 1997; Bach and Dolan, 2012). The biological basis of these inter-individual differences are much less studied and understood compared to the case for contextual modulators of attitude toward risk.

Previous research in behavioral genetics has shown that inter-individual variance in risk attitude may be highly heritable. Measures of risk taking were more correlated among monozygotic versus dizygotic twins and overall, the influence of shared genes explained about 20% of the variance in risky behavior (Cesarini et al., 2009). A similar figure around 20% was suggested for the genetic influence on risk attitude by another study that examined the relationship between polymorphism in Dopamine D4 receptor and financial risk taking in men (Dreber et al., 2009). More recently it has also been observed that carriers of a certain L polymorphism of monoamine oxidase-A were more accurate in their risk assessment and more willing to take financial risks (Frydman et al., 2011). These findings linking central nervous system neurotransmitter mechanisms to trait-like variations in risk attitude raise the question whether variations in brain structure may also contribute to inter-individual differences in risky behavior (Kanai and Rees, 2011).

In recent years there have been many studies on functional neural correlation of risk taking behavior in disorders such as anxiety, attention deficits, pathological gambling, and substance abuse (Martins et al., 2004; Singer et al., 2004; Kathleen Holmes et al., 2009). Evidence from neuropsychological, neuroimaging, and animal studies suggest that decision making under risk involves a network of cortical and subcortical regions including orbitofrontal cortex (OFC), dorsolateral prefrontal cortex (DLPFC), parietal cortices, and caudate, anterior cingulate cortex (ACC) and thalamus (Ernst and Paulus, 2005; Trepel et al., 2005; Krain et al., 2006). However, which of these neuronal structures contributes to individual differences in risk taking is unknown.

Balloon analog risk task (BART; Lejuez et al., 2002; see below for a description) has been extensively used to study the neuronal substrates of risk taking behavior in human. In this task the subject is asked to repeatedly choose whether or not to inflate a balloon that could expand or explode as a consequence of the subject's choice to inflate. The larger the balloon sizes the higher the probability of the explosion as well as the larger the collectable reward if the explosion is avoided. Thus, this task is a model of real-world situation in which taking a new risk gives one a probabilistic chance to increase profits at the expense of accepting a probability for losing what one already has (e.g., health, safety, savings, etc.) (Lejuez et al., 2002, 2003a,b, 2007; Hunt et al., 2005). Higher BART scores indicate more risk seeking. A fMRI study using BART revealed activations in the mesolimbic-frontal regions, including the midbrain, ventral and dorsal striatum, anterior insula (AI), dorsal lateral prefrontal cortex (DLPFC), and anterior cingulate/medial frontal cortex (ACC/MFC) (Rao et al., 2008). Results from patient studies using the same task also accorded with the neuroimaging findings.

In one recent fMRI study using the BART, (Fukunaga et al., 2012), the authors looked for distinct neural correlations of loss aversion and reward seeking signals. They hypothesized that two brain areas, anterior insula (AI) and inferior frontal gyrus (IFG) are involved in the loss aversion independent of reward seeking or the expectedness of reward and punishment. A modified version of BART was used to test this hypothesis. The activity at the ACC and AI/IFG were increased when participants chose to discontinue inflating the balloon. This finding accorded well with increasing loss aversion. In addition, a significant activation was observed in the vmPFC area associated with subjects continuing to inflate the balloon (i.e., when they decided to take a risk rather than collect the accumulated reward), which was also in agreement with previous findings that implicated vmPFC in reward seeking.

Putting the above findings together, we asked whether the individual variability in risk taking behavior as measured by BART may have specific correlations in the gray matter structure of the human brain. Based on the previous fMRI works (Rao et al., 2008; Fukunaga et al., 2012; Canessa et al., 2013) we hypothesized that the brain regions AI/IFG, ACC, vmPFC, DLPFC, posterior insula and Amygdala are most likely to reflect the individual differences in risk taking in the context of BART. We conducted a Voxel Based Morphometry study (Ashburner and Friston, 2000, 2005; Ashburner, 2007) to assess whether a gray matter structure in a set of previously identified regions of interest is correlated with the BART score.

## Method

Fifty two healthy college students, 30 males, 22 females ranging from 20 to 32 years old with the average (μ) age of 21.9, and the standard deviation (σ) of 1.76 participated in the study. They were recruited by advertisements placed in the Shahid Beheshti University of Medical Science campus and its nearby community. The participants did not report any illnesses in the past as well as at the time of evaluation, and neither did use any psychoactive medications. The experiments were approved by the ethical committee in the Shahid Beheshti medical school, and all the participants were given a written informed consent.

### Behavioral measurement of risk attitudes

#### Stimuli and procedure

**Figure 1:**
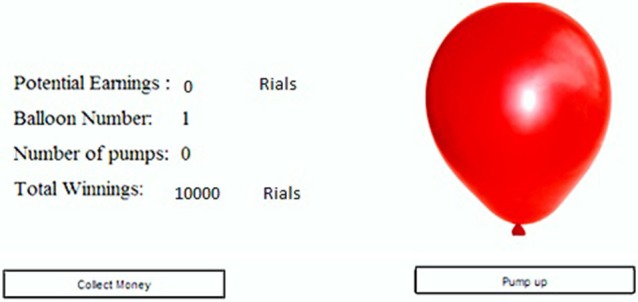
**Display panel in the BART**. The balloon was expanded by clicking on the “Pump up” button. Each click added 100 Rials (equivalent to 10¢) to the “potential earnings.” By clicking on “collect money” button, the money accumulated thus far in “Potential Earnings” was transferred to “Total winnings.” If the balloon is exploded, the money in the “potential earnings” was subtracted from the “Total winnings.” The “Balloon number” indicates the trial number.

We used a version of BART (Rao et al., 2008) that was modified from the original BART (Lejuez et al., 2002) (Figure 1) in rewarding and punishing the participants and also in calculating BART scores. In modified BART a realistic image of a balloon was presented to the participants in the center of the screen in a standard illuminated room. They were instructed to choose between inflating the balloon by pressing “pump up” button repeatedly or stop and harvest the collected reward by pressing the “collect the money” button. They were also informed that the balloon could explode at any size but the probability of explosion associated with every pump was not mentioned.

Each participant started the experiment with an endowment of 10000 Rials (subjectively equivalent to 10 $). At the beginning of each trial, a small balloon (4 cm in its biggest diameter, 50 cm distance from the screen) was displayed on the computer monitor (Figure 1) and the participants chose to either “Pump Up” or “Collect the Money” by the mouse click. If the participant chose to pump up, the balloon might explode with some probability; this is explained in the following paragraph. If the balloon did not explode, then the participant's earnings from the current trial was increased by 100 Rials (subjectively equivalent to 10¢). If the balloon exploded or the participant chose to collect the money, then the trial was terminated and the money was added to her/his endowment. In the case of explosion, the current trial's earnings prior to explosion were deducted from the participant's total endowment. Each participant was supposed to complete 30 trials.

The probability that a balloon would explode was 1/128 for the first pump. If the balloon did not explode after the first pump, explosion probability for the second pump is altered to 1/127, for the third pump, it is 1/126. This trend goes on until; at the 128th pump the probability of the explosion reaches unity (i.e., 1/1 or 100%). This distribution of explosion probabilities means that if the participant never chooses to collect the money, on average, the explosion occurs at the 64th pump-up. As pointed out in the literature (Lejuez et al., 2002), this task is a laboratory model of real-world situations in which taking excessive risk often decreases the outcome, and jeopardizes one's health, safety and property. Each successful pump-up (i.e., not followed by an explosion) increases the temporary earning which could be lost in a future explosion (contributing to a greater future regret) and decreases the relative gain from future pump-ups (diminishing the prospect of further risk). For example, after a successful first pump-up, the next pump risk losing only the 100 Rials earned so far. The second pump-up would increase the possible earnings by 100%; however, the 31st pump-up would only increase the current earnings of the trial by 3.3% (i.e., from 3000 Rials to 3100 Rials). Note that the participants were unaware of maximum number of inflations and the probability distribution of the explosions.

#### Data analysis

There are several ways to measure the BART score. We utilized the convention that could help us quantifying the participant's attitude toward the risk in the most accurate way. We used the adjusted score, i.e., average number of pumps during the task except the trials terminated by explosion. This adjusted score is preferred to the unadjusted score which also included the exploded balloons because the number of pumps depends on the exploded balloons, which limits between-participant-difference in the unadjusted score (Lejuez et al., 2002). Behavioral studies (Lejuez et al., 2002, 2003a,b, 2007; Hunt et al., 2005) have shown that the participants' BART scores correlate with the scores on a number of real-world risk attitude measures such as sensation seeking and impulsivity questionnaires as well as the occurrence of risky behaviors (e.g., drug addiction, smoking and delinquency). All behavioral analyses were done in SPSS17.0 software. (http://www.spss.com).

#### Structural imaging

A Siemens MAGNETOM Avanto 1.5 Tesla MRI machine with a standard quadrature head coil were used for the structural brain imaging. T1-weighted images (160 contiguous, 1-mm thick, *TE* = 3.5 ms, *TR* = 12 ms axial slices) were generated. We used Statistical Parametric Mapping 8 (SPM8) (http://www.fil.ion.ucl.ac.uk/spm) to do Voxel Based Morphometry analysis (Ashburner and Friston, 2000, 2005; Ashburner, 2007).

All scans were manually reoriented and realigned with respect to the anterior commissure. Using SPM8 software,T1 images were segmented into gray matter, white matter and cerebrospinal fluid. We then used Diffeomorphic Anatomical Registration integrated in the Exponentiated Lie algebra (DARTEL) to build the gray matter template of each participant for registration. The images then were smoothed using a Gaussian kernel with an 8 mm full-width half-maximum, and finally were normalized into Montreal Neurological Institute (MNI) space.

For statistical analysis we used a General Linear Model approach described in (Mccullagh and Nelder, 1989). This method looks at the correlation between the gray matter volume and the behavioral performance in BART. Our design matrix consist of BART scores as the covariate of interest, and age, gender and total gray matter volume as covariates of no interest. We employed multiple comparison theory (Hsu, 1996) to adjust our statistics.

In order to test our anatomically circumscribed hypothesis we restricted our analysis to predefined regions of interest and used small volume correction to test whether the gray matter structure in those regions were correlated with BART score or not. We defined our regions of interest (ROIs) according to the literature (Rao et al., 2008; Fukunaga et al., 2012) ACC, IFG/AI, vmPFC and DLPFC. To test, we performed an ROI analysis on the coordinates derived from the previous studies (see Table 1). A sphere of 10 mm diameter was made around each coordinate with *p*-value (uncorrected) < 0.001. Another ROI analysis was also done with constructing a union image of all the mentioned areas using MarsBar toolbox of SPM8 (http://marsbar.sourceforge.net).

**Table 1:** **Candidate brain areas, Talairac coordinates and the reference articles linking them to BART score**.

**Brain area**	**MNI Coordinates (reference article)**
ACC	Right: 6, 26, 24 (Fukunaga et al., 2012) Bilateral: 0, 12, 42 (Rao et al., 2008)
rAI/IFG	48, 20, −6 (Fukunaga et al., 2012)
lAI/IFG	−4, 16, −8 (Fukunaga et al., 2012)
vmPFC	Left: −12, 36–18 (Fukunaga et al., 2012)
DLPFC	Right: 30, 36, 20 (Rao et al., 2008) Left: −34, 46, 16 (Rao et al., 2008)

The results are reported in the Talairach coordinate using GingerALE software (http://www.brainmap.org/ale/index.html) and anatomical locations were determined by Talairach Client (http://www.talairach.org/client.html) (Lancaster et al., 1997, 2000).

## Results

**Figure 2:**
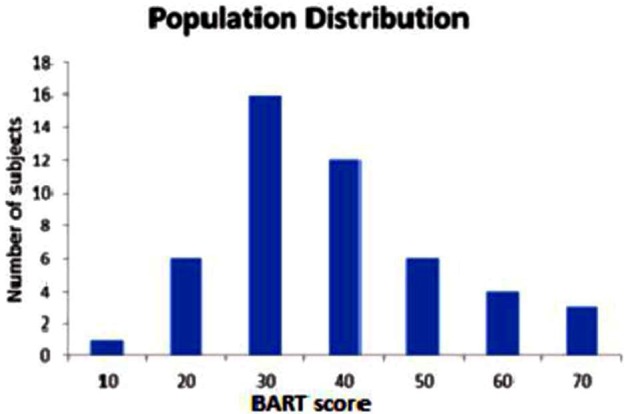
**Measured BART score of 48 participants**. (Mean = 33.50, Standard Deviation = 13.19, variance = 174.12) The frequency histogram shows the number of participants as a function of BART score.

Four participants were removed from the study because they produced extreme outlier negative BART scores which were greater than 2 S.D.S from population mean. The included participants consisted of 28 males and 20 females with the ages ranging from 20 to 30, with the average age of 21.9. The BART scores (mean score = 33.50; Standard deviation = 14.29; see Figure 2) showed a normal distribution with the following parameters (*Z* = 0.712, *p* = 0.691) in Kolmogorov–Smirnov test.

**Figure 3:**
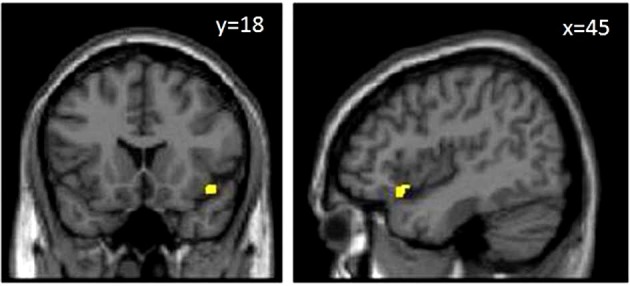
**MRI T1 weighted images**. The yellow area in the images above shows anterior insula. Results are represented with *p* < 0.05 with multiple comparison correction at cluster level with an underlying *p* < 0.001 uncorrected on voxel–level.

**Figure 4:**
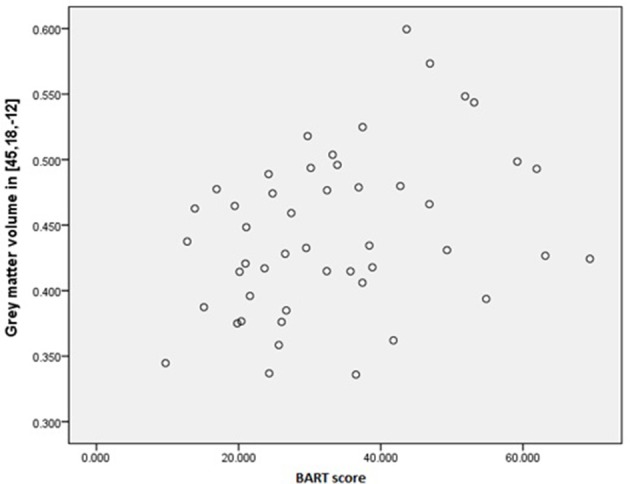
**Interaction between AI and BART results**. Positive correlation between Anterior Insula (AI), and the BART results (MNI coordinate [45 18 −12]).

We assessed predefined areas that showed positive or negative correlations between the size of the gray matter volume, and the BART performance to make a unified image using MarsBar toolbox of SPM. The results of the ROI analysis for the unified image was not significant (*p* = 0.10). Moreover, the results of one by one ROI analysis revealed that there was a significant positive correlation between Anterior Insula (AI), and the BART results (MNI coordinate [45 18 −12], *P*(FWE-cor) = 0.03, *T* = 3.53, Cluster extend = 11) (Figures 3, 4). The region of interest in this area was [48 20 -6] MNI coordinate (Fukunaga et al., 2012). We did not observe any brain region to have reached a statistical significance for the negative correlation in the small volume correction analysis (*P* < 0.05, FWE-corrected).

## Discussion

The research question was whether inter-individual variability in risk preference could be related to inter-individual variability in brain structures. Based on the previous neuroimaging findings (Kuhnen and Knutson, 2005; van ‘t Wout et al., 2005; Fecteau et al., 2007; Rao et al., 2008; Strenziok et al., 2011; Fukunaga et al., 2012), we hypothesized a number of brain areas (see Table 1) such as the anterior insula (Tversky, 1992), ACC, vmPFC and DLPFC would be candidates for showing the expected relationship. Our findings showed that among the candidate areas, there is only a significant positive correlation in the anterior insula, between gray matter volume and the obtained BART score.

This brings up the important question of what special role the anterior insula may play in risk taking behavior. This is important as to what characteristics of the BART are correlated better with the risk taking behavior associated with a given brain area. It will be helpful to note that the different roles of anterior insula includes risk prediction error (Preuschoff et al., 2008; Bossaerts, 2010) as well as contribution to the representation of objective versus subjective value of rewards (Schultz, 2010). One hypothesis connecting our findings with the previous results is that the subjects with higher BART scores (with correspondingly larger gray matter volume in the anterior insula) underestimate the reward prediction error associated with a possible bubble burst and thereby overweight the probability of getting the reward compared to less risk-taking subjects who scored lower in the BART (and had smaller size of the gray matter volume in anterior insula). Future, computational modeling analysis of the behavior in BART task is required to test this prediction.

Another way to interpret the results is to consider a particular feature of the BART task. Every choice to pump up is likely to lead to either a small increment in the reward or a devastating total loss. We evaluate the rewards expected from our choices based on objective or subjective values (Schultz, 2010). The objective value of a reward is the real monetary amount of the reward whereas its subjective value is influenced by particular characteristics of an individual such as his/her inborn or acquired attitude and beliefs. It has been argued that the anterior insula makes a crucial contribution to the formation of our subjective experience (Craig, 2009). Putting these ideas together it leads to the suggestion that people with larger size of the gray matter volume may assign a higher subjective value to rewards leading to a more risky behavior and higher BART scores.

It is interesting that while the coordinate [48 20 −6] is highly associated with areas Insula (Z-score = 7.1), AI (Z-score = 6.91) and frontal operculum (Z-score = 6.98), (all derived from http://www.neurosynth.org), the coordinate [45 18 −12] specified in our results is associated to the area IFG (Z-score = 3.7), known to play a role in cognitive control as well as AI (Z-score = 4.2). This however could be important when interpreting data in a cognitive control task.

In addition to fMRI studies, there are some VBM and structural studies done on risk taking. As far as we know this is the first VBM study in which we use BART as a paradigm case of a risk taking behavior. Other studies use other paradigms such as Cambridge gamble task or Cups task in some lesion study tasks (Clark et al., 2008; Weller et al., 2009). Although, interesting effects should not be task dependent, it is important that there are as yet only a few experiments based on structural methods reported in the literature. It is therefore not possible at this early stage to judge what differences matter most in the reported results. For example results reported by Canessa et al. (2013) is not only based on a different task, but the authors further point out the role of posterior insula area in risk taking behavior and do not specifically maintain the anterior insula region which is relevant to our data. Also the lesion studies (Clark et al., 2008; Weller et al., 2009) seem to imply different roles for the insular cortex. In lesion study done by Clark et al. (2008) four groups of participants including vmPFC lesion, insular lesion, healthy subjects and control lesion affecting dorsal and lateral frontal cortex were studied under Cambridge gamble task to differentiate distinctive roles of vmPFC and Insula roles in risk taking behavior. Results showed increased betting regardless of the odds of winning in patients with vmPFC damage. In contrast patients with insular cortex lesion failed in adjusting odds of winning which the authors consider it as consistent with the role of Insula in loss aversion. In another study (Weller et al., 2009), patients with focal insular lesion and healthy individuals are compared on risk taking in terms of achieving gains and avoiding losses. It was revealed that lesion patients made fewer risky choices than healthy adults in gain domain. All together, future investigation is required to understand the specific commonalities and differences between these paradigms and their neuronal substrates. There are also other functional imaging modalities that are recently used for the brain (Nasiriavanaki et al., 2014) which with their higher resolution capability may provide us with more details.

## Conclusion

We asked a question whether local structural variations in the human brain are related to individual differences in risk taking behavior. To answer this, we used Balloon analog risk task as a behavioral measure. Voxel based Morphometry was done on T1-weighted images of 48 subjects. Results of region of interest analysis revealed that among all areas candidate for risk taking behavior, there is a significant positive correlation between anterior insula and BART results.

### Conflict of interest statement

The authors declare that the research was conducted in the absence of any commercial or financial relationships that could be construed as a potential conflict of interest.

## References

[B1] Ashburner J. (2007). A fast diffeomorphic image registration algorithm. Neuroimage 38, 95–113. 10.1016/j.neuroimage.2007.07.007 17761438

[B2] Ashburner J. Friston K. J. (2000). Voxel-based morphometry–the methods. Neuroimage 11, 805–821. 10.1006/nimg.2000.0582 10860804

[B3] Ashburner J. Friston K. J. (2005). Unified segmentation. Neuroimage 26, 839–851. 10.1016/j.neuroimage.2005.02.018 15955494

[B4] Bach D. R. Dolan R. J. (2012). Knowing how much you don't know: a neural organization of uncertainty estimates. Nat. Rev. Neurosci. 13, 572–586. 10.1038/nrn3289nrn3289 22781958

[B5] Bossaerts P. (2010). Risk and risk prediction error signals in anterior insula. Brain Struct. Funct. 214, 645–653. 10.1007/s00429-010-0253-1 20512378

[B6] Canessa N. Crespi C. Motterlini M. Baud-Bovy G. Chierchia G. Pantaleo G.. (2013). The functional and structural neural basis of individual differences in loss aversion. J. Neurosci. 33, 14307–14317. 10.1523/JNEUROSCI.0497-13.2013 24005284 PMC6618376

[B7] Cesarini D. Dawes C. T. Johannesson M. Lichtenstein P. Wallace B. (2009). Genetic Variation in Preferences for Giving and Risk Taking. Q. J. Econ. 124, 809–842. 10.1162/qjec.2009.124.2.809

[B8] Clark L. Bechara A. Damasio H. Aitken M. R. Sahakian B. J. Robbins T. W. (2008). Differential effects of insular and ventromedial prefrontal cortex lesions on risky decision-making. Brain 131, 1311–1322. 10.1093/brain/awn066 18390562 PMC2367692

[B9] Craig A. D. (2009). How do you feel–now? The anterior insula and human awareness. Nat. Rev. Neurosci. 10, 59–70. 10.1038/nrn2555nrn2555 19096369

[B10] Damasio A. R. (1996). The somatic marker hypothesis and the possible functions of the prefrontal cortex. Philos. Trans. R. Soc. Lond., B. Biol. Sci. 351, 1413–1420. 10.1098/rstb.1996.0125 8941953

[B11] De Martino B. Kumaran D. Seymour B. Dolan R. J. (2006). Frames, biases, and rational decision-making in the human brain. Science 313, 684–687. 10.1126/science.1128356 16888142 PMC2631940

[B12] Dreber A. Address C. L. A. Eisenberg D. T. A. Garcia J. R. Zamore R. S. Lum J. K. (2009). The 7R polymorphism in the dopamine receptor D4 gene (DRD4) is associated with financial risk taking in men. Evol. Hum. Behav. 30, 85–92. 10.1016/j.evolhumbehav.2008.11.001

[B13] Elke U. Weber R. A. M. (1997). Perceived risk attitudes: relating risk perception to risky choice. Manage. Sci. 43, 123–144.

[B14] Ernst M. Paulus M. P. (2005). Neurobiology of decision making: a selective review from a neurocognitive and clinical perspective. Biol. Psychiatry 58, 597–604. 10.1016/j.biopsych.2005.06.004 16095567

[B15] Fecteau S. Pascual-Leone A. Zald D. H. Liguori P. Théoret H. Boggio P. S.. (2007). Activation of prefrontal cortex by transcranial direct current stimulation reduces appetite for risk during ambiguous decision making. J. Neurosci. 27, 6212–6218. 10.1523/JNEUROSCI.0314-07.2007 17553993 PMC6672163

[B16] Fleming S. M. Thomas C. L. Dolan R. J. (2010). Overcoming status quo bias in the human brain. Proc. Natl. Acad. Sci. U.S.A. 107, 6005–6009. [pii] 10.1073/pnas.09103801070910380107 20231462 PMC2851882

[B17] Frydman C. Camerer C. Bossaerts P. Rangel A. (2011). MAOA-L carriers are better at making optimal financial decisions under risk. Proc. Biol. Sci. 278, 2053–2059. 10.1098/rspb.2010.2304 21147794 PMC3107654

[B18] Fukunaga R. Brown J. W. Bogg T. (2012). Decision making in the Balloon Analogue Risk Task (BART): anterior cingulate cortex signals loss aversion but not the infrequency of risky choices. Cogn. Affect. Behav. Neurosci. 12, 479–490. 10.3758/s13415-012-0102-1 22707378 PMC3493559

[B19] Hsu J. (1996). Multiple Comparisons: Theory and Methods (Guilford School Practitioner). Boca Raton, FL: Chapman and Hall/CRC press.

[B20] Hunt M. K. Hopko D. R. Bare R. Lejuez C. W. Robinson E. V. (2005). Construct validity of the Balloon Analog Risk Task (BART): associations with psychopathy and impulsivity. Assessment 12, 416–428. 10.1177/1073191105278740 16244122

[B21] Jentsch J. D. Woods J. A. Groman S. M. Seu E. (2010). Behavioral characteristics and neural mechanisms mediating performance in a rodent version of the Balloon Analog Risk Task. Neuropsychopharmacology 35, 1797–1806. 10.1038/npp.2010.47 20375994 PMC3055471

[B22] Kanai R. Rees G. (2011). The structural basis of inter-individual differences in human behaviour and cognition. Nat. Rev. Neurosci. 12, 231–242. 10.1038/nrn3000 21407245

[B23] Kathleen Holmes M. Bearden C. E. Barguil M. Fonseca M. Serap Monkul E. Nery F. G.. (2009). Conceptualizing impulsivity and risk taking in bipolar disorder: importance of history of alcohol abuse. Bipolar Disord. 11, 33–40. 10.1111/j.1399-5618.2008.00657.x 19133964 PMC4187105

[B24] Krain A. L. Wilson A. M. Arbuckle R. Castellanos F. X. Milham M. P. (2006). Distinct neural mechanisms of risk and ambiguity: a meta-analysis of decision-making. Neuroimage 32, 477–484. 10.1016/j.neuroimage.2006.02.047 16632383

[B25] Kuhnen C. M. Knutson B. (2005). The neural basis of financial risk taking. Neuron 47, 763–770. 10.1016/j.neuron.2005.08.008 16129404

[B26] Lancaster J. L. Rainey L. H. Summerlin J. L. Freitas C. S. Fox P. T. Evans A. C.. (1997). Automated labeling of the human brain: a preliminary report on the development and evaluation of a forward-transform method. Hum. Brain Mapp. 5, 238–242. 10.1002/(SICI)1097-0193(1997)5:4&lt;238::AID-HBM6&gt;3.0.CO;2-4 20408222 PMC2860189

[B27] Lancaster J. L. Woldorff M. G. Parsons L. M. Liotti M. Freitas C. S. Rainey L.. (2000). Automated Talairach atlas labels for functional brain mapping. Hum. Brain Mapp. 10, 120–131. 10.1002/1097-0193(200007)10:3<120::AID-HBM30>3.0.CO;2-8 10912591 PMC6871915

[B28] Lejuez C. W. Aklin W. Daughters S. Zvolensky M. Kahler C. Gwadz M. (2007). Reliability and validity of the youth version of the Balloon Analogue Risk Task (BART-Y) in the assessment of risk-taking behavior among inner-city adolescents. J. Clin. Child Adolesc. Psychol. 36, 106–111. 10.1080/15374410709336573 17206886

[B29] Lejuez C. W. Aklin W. M. Jones H. A. Richards J. B. Strong D. R. Kahler C. W.. (2003a). The Balloon Analogue Risk Task (BART) differentiates smokers and nonsmokers. Exp. Clin. Psychopharmacol. 11, 26–33. 10.1037/1064-1297.11.1.26 12622341

[B30] Lejuez C. W. Aklin W. M. Zvolensky M. J. Pedulla C. M. (2003b). Evaluation of the Balloon Analogue Risk Task (BART) as a predictor of adolescent real-world risk-taking behaviours. J. Adolesc. 26, 475–479. 10.1016/S0140-1971(03)00036-8 12887935

[B31] Lejuez C. W. Read J. P. Kahler C. W. Richards J. B. Ramsey S. E. Stuart G. L.. (2002). Evaluation of a behavioral measure of risk taking: the Balloon Analogue Risk Task (BART). J. Exp. Psychol. Appl. 8, 75–84. 10.1037/1076-898X.8.2.75 12075692

[B32] Martins S. S. Tavares H. da Silva Lobo D. S. Galetti A. M. Gentil V. (2004). Pathological gambling, gender, and risk-taking behaviors. Addict. Behav. 29, 1231–1235. 10.1016/j.addbeh.2004.03.023 15236828

[B33] McCullagh P. Nelder J. A. (1989). Generalized Linear Models, Chapman & Hall/CRC Monographs on Statistics & Applied Probability, 2nd Edn., Vol. 37 Boca Raton, FL: Chapman and Hall/CRC press.

[B34] Nasiriavanaki M. Xia J. Wan H. Bauer A. Q. Culver J. P. Wang L. V. (2014). High-resolution photoacoustic tomography of resting-state functional connectivity in the mouse brain. Proc. Natl. Acad. Sci. USA 111, 21–26. 10.1073/pnas.1311868111 24367107 PMC3890828

[B35] Preuschoff K. Quartz S. R. Bossaerts P. (2008). Human insula activation reflects risk prediction errors as well as risk. J. Neurosci. 28, 2745–2752. 10.1523/JNEUROSCI.4286-07.2008 18337404 PMC6670675

[B36] Rao H. Korczykowski M. Pluta J. Hoang A. Detre J. A. (2008). Neural correlates of voluntary and involuntary risk taking in the human brain: an fMRI Study of the Balloon Analog Risk Task (BART). Neuroimage 42, 902–910. 10.1016/j.neuroimage.2008.05.046 18582578 PMC9475445

[B37] Schultz W. (2010). Subjective neuronal coding of reward: temporal value discounting and risk. Eur. J. Neurosci. 31, 2124–2135. 10.1111/j.1460-9568.2010.07282.x 20497474

[B38] Singer T. Seymour B. O'doherty J. Kaube H. Dolan R. J. Frith C. D. (2004). Empathy for pain involves the affective but not sensory components of pain. Science 303, 1157–1162. 10.1126/science.1093535 14976305

[B39] Strenziok M. Pulaski S. Krueger F. Zamboni G. Clawson D. Grafman J. (2011). Regional brain atrophy and impaired decision making on the balloon analog risk task in behavioral variant frontotemporal dementia. Cogn. Behav. Neurol. 24, 59–67. 10.1097/WNN.0b013e3182255a7c 21697710

[B40] Trepel C. Fox C. R. Poldrack R. A. (2005). Prospect theory on the brain? Toward a cognitive neuroscience of decision under risk. Brain Res. Cogn. Brain Res. 23, 34–50. 10.1016/j.cogbrainres.2005.01.016 15795132

[B41] Tversky A. Kahneman D. (1981). The framing of decisions and the psychology of choice. Science 211, 453–458. 7455683 10.1126/science.7455683

[B42] Tversky D. K. A. (1979). Prospect theory: an analysis of decision under risk. Econometrica 47, 263–292.

[B43] Tversky K. (1992). Advances in prospect theory: cumulative representation of uncertainty. Risk Uncertain. 5, 26 10.1007/BF00122574

[B44] van ‘t Wout M. Kahn R. S. Sanfey A. G. Aleman A. (2005). Repetitive transcranial magnetic stimulation over the right dorsolateral prefrontal cortex affects strategic decision-making. Neuroreport 16, 1849–1852. 10.1097/01.wnr.0000183907.08149.14 16237340

[B45] Weller J. A. Levin I. P. Shiv B. Bechara A. (2009). The effects of insula damage on decision-making for risky gains and losses. Soc. Neurosci. 4, 347–358. 10.1080/17470910902934400 19466680

